# Defining “Hyperacute T Waves Without STEMI” as a Special Entity of Occlusion Myocardial Infarction Prone to Worse Outcomes

**DOI:** 10.1016/j.jacadv.2025.102342

**Published:** 2025-11-13

**Authors:** Yehya Khlidj, Mourad Boukheloua, Anwar Chahal

**Affiliations:** a Faculty of Medicine, University of Algiers 1, Algiers, Algeria; b Department of Biology, University of Sciences and Technology Houari Boumediene, Algiers, Algeria; c Cardiology Department. Nafissa Hammoud University Hospital, Algiers, Algeria; d Department of Cardiology, Barts Heart Centre, London, UK; e Northumbria Hospitals NHS Foundation Trust, Newcastle Upon Tyne, United Kingdom; f William Harvey Research Institute, Queen Mary University of London, London, United Kingdom; g Center for Inherited Cardiovascular Diseases, Department of Cardiology, WellSpan Health, York, Pennsylvania, USA

Using anomalies in T waves magnitude and symmetry, Pendell Meyers et al brilliantly designed a software-calculated score for the detection of occlusion myocardial infarction (OMI). Impressively, the hyperacute T waves (HATW) defined by this score were found to be specific to OMI even in the absence of traditional STEMI criteria.^1^ Such findings further solidify the high value of HATW in the setting of clinicobiological suspicion of acute coronary syndrome. Thus, in this context, HATW alone constitute an STEMI equivalent that can independently justify emergent PCI without the need for serial ECGs/troponins and regardless of the classic risk scores (ie, TIMI and GRACE).^2^

What was particularly noticeable for us in the study is that about 3% (49% in a different study^3^) of the cohort had HATW without STEMI, the vast majority of them displayed angiographically confirmed OMI. Moreover, in 42% of patients with OMI exhibiting isolated HATW on ECG, an angiogram was not performed within the first 90 minutes as it is recommended. This could be due to HATW being perceived as an early subclinical or borderline sign in the dynamic ECG changes that will eventually evolve into STEMI, where cardiologists may think less invasive is the correct timely approach. HATW without STEMI patients are therefore prone to delayed reperfusion but also increased mortality.^3^ We believe that defining this group and separating it from the STEMI’s would serve as a beneficial step to optimize the outcomes of OMI and promote earlier primary PCIs.

**Figure 1:**
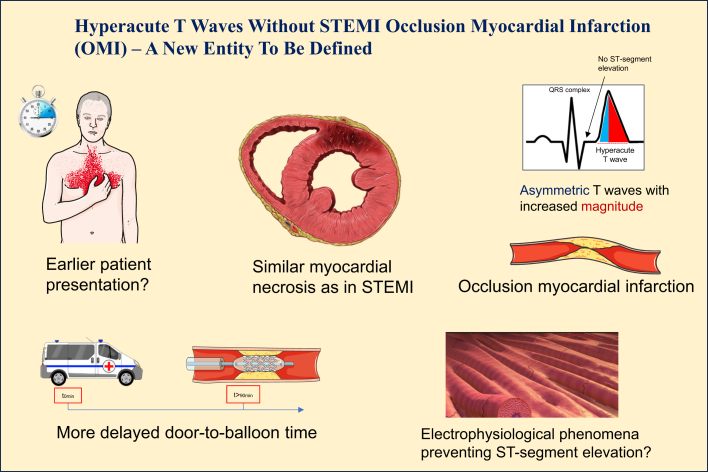
**Characterizing Features of Hyperacute T Waves (HATW) Without STEMI Entity in Occlusion Myocardial Infarction (OMI) Patients** In this entity, patients present with a clinicobiological context of acute coronary syndrome perhaps earlier than usual however their ECG shows HATW without ST-segment elevation criteria that fulfill STEMI diagnosis. Due to the absence of STEMI, an invasive revascularization approach may be delayed favoring extended necrosis and worse mortality. On angiography, the culprit lesion is completely occluded while analysis of the concerned myocardium reveals areas of necrosis comparable to those observed in STEMI. Unique unknown mechanisms appear to induce persistence of HATW while preventing electrophysiological modifications that give rise to the classic ST-segment elevation.

Electrophysiologically speaking, the time from the genesis of HATW to ST-segment elevation is typically short, within minutes following the rapid transition of the endo-epicardial voltage gradient of repolarization. Whereas the persistence of HATW was previously thought to reflect protective mechanisms like collateral circulation that modulate ischemia-induced electrical changes, thus preventing ST-segment elevation occurrence. However, this notion has been questioned, and it was shown that it is rather a matter of different distribution of necrosis wave-front across myocardium with comparable tissular injury as seen during STEMI.^4^ This suggests possible unique pathophysiological features in HATW without STEMI patients, further supporting their distinctive OMI phenotype (Figure 1).
